# Open questions in enterovirus uncoating

**DOI:** 10.1128/jvi.01966-25

**Published:** 2026-05-15

**Authors:** Walker Symonds-Orr, Patrick T. Dolan

**Affiliations:** 1 Quantitative Virology and Evolution Unit, Laboratory of Viral Diseases, NIH-NIAID Division of Intramural Research 469049 , Bethesda, Maryland, USA; 2 Biological Sciences Graduate Program, University of Maryland 1068 , College Park, Maryland, USA; Indiana University Bloomington, Bloomington, Indiana, USA

**Keywords:** enteroviruses, virus entry, endosomes, virion structure, picornavirus

## Abstract

Enterovirus uncoating, the events leading to the release of the viral genome into the cytoplasm, remains a poorly understood process. This review discusses two competing models: the “genome translocation model,” where viral proteins form a pore for RNA passage into the cytosol, and the “particle escape model,” where the entire virion crosses the endosomal membrane before uncoating. We discuss the combination of structural biology, *in vitro* reconstitution, and cellular assays required to definitively resolve this long-standing mystery.

## INTRODUCTION

Enveloped viruses release their genomes into the cytosol of susceptible cells by membrane fusion, a well-characterized process essential to multiple cellular functions. How non-enveloped viruses, like picornaviruses, overcome this topological problem is, by contrast, poorly understood. Enteroviruses are a genus of picornavirus with an icosahedral capsid enclosing a 7–7.5 kb positive-sense single-stranded genome. The genus *Enterovirus* comprises four species of human-infecting enterovirus, A–D, and three species of human rhinovirus, A–C. Collectively, over 300 antigenically distinct types have been identified (1). These viruses cause a wide diversity of human diseases (2, 3). The most well-studied enterovirus is poliovirus, and it has served as the prototype for studies on enterovirus entry and uncoating.

## ENTEROVIRUS VIRION BIOGENESIS AND ASSEMBLY

**Fig 1:**
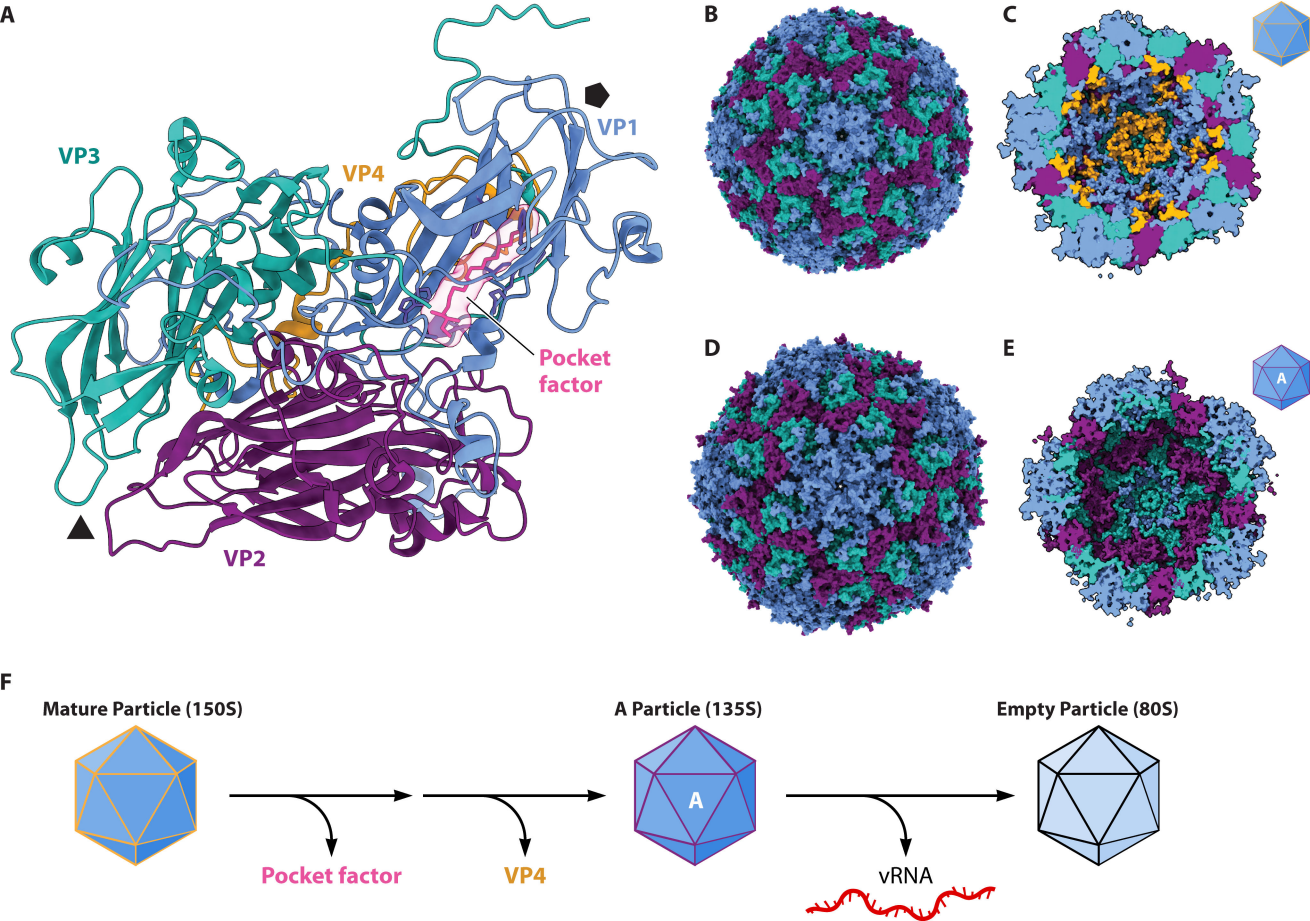
Structure of the enterovirus mature capsid and A-particle. (**A**) *Enterovirus* A71 capsid proteins from mature virions with “pocket factor” lipid shown in pink. VP1, blue; VP2, purple; VP3, green; VP4, yellow. The pentagon denotes a fivefold axis, and the triangle denotes a threefold axis. (**B**) Structure of *Enterovirus* A71 mature virion capsid (RNA not shown); PDB: 8E2X. (**C**) Slice of the internal surface of the capsid focusing on a single pentamer. Note VP4 lining the inside of the capsid around the fivefold axes of symmetry. (**D**) Structure of *Enterovirus* A71 A-particle following loss of pocket factor and VP4; PDB: 8E2Y. (**E**) Slice showing the internal face with VP4 absent. (**F**) Schematic showing the transition from an infectious virion to an empty particle and the corresponding loss of material.

The enterovirus virion assumes a number of characteristic metastable states at different points of the life cycle (Fig. 1A), which are referred to by their sedimentation coefficients. These include the “immature particle,” “mature virion” (160S for poliovirus and 150S for many other enteroviruses), “A-particle” (135S), and “empty capsid” (80S) (Fig. 1B). Virion biogenesis begins in an infected cell when 60 copies of a heterotrimeric assembly of capsid proteins, VP0, VP1, and VP3, assemble together with a molecule of genomic (positive-sense) viral RNA. The viral RNA is conjugated at the 5′ end to the viral protein 3B (VPg). Empty capsids composed of VP0, VP1, and VP3 are also produced but are not discussed further. The capsid proteins originate as products of the viral polyprotein, resulting from cleavage by the viral proteases 3CD and 3C (4). During assembly, each VP1 incorporates a lipid molecule called a “pocket factor,” typically modeled as sphingosine, palmitate, stearate, or other shorter-chain fatty acids, into a hydrophobic pocket (Fig. 1A) (5). The hydrophobic pocket is also the target of drugs that stabilize the virion and prevent uncoating (6–9). Certain enteroviruses with respiratory transmission, including some genotypes of EV-D68 and human rhinovirus types 3 and 14, do not appear to incorporate a pocket factor (10–15). The causes and consequences of the divergence in this structural trait are not understood.

The *“*immature virion” is not infectious. In a poorly understood process called “maturation,” VP0 is cleaved into VP2 and the N-terminally myristoylated VP4, yielding the infectious mature virion (16, 17). This process requires packaged RNA and requires acidic cellular compartments in poliovirus and EV-D68 (18, 19). In resolved structures of the mature virion, VP4 lies entirely on the inside of the capsid (20–22). Virion biogenesis and maturation are reviewed in reference 23; the viral life cycle is reviewed in reference 24.

## ENTRY AND TRAFFICKING

The entry process begins with adsorption to the surface of a cell mediated by the viral uncoating receptor or by attachment factors such as glycosylated and sulfated molecules (25–29). The requirement for or benefit from the use of additional attachment factors may be specific to cell type and context. Substitutions increasing attachment to glycans or heparan sulfates are common cell culture adaptations in many enteroviruses; in EV-A71, these mutations are attenuating *in vivo* (30–34). After adsorption, the virus is internalized from the cell surface and trafficked to compartments that support uncoating. For most enteroviruses, there is no evidence for uncoating at the plasma membrane, although it is technically possible that poliovirus uncoating takes place from sealed invaginations without further trafficking (35). Ectopic uncoating at the plasma membrane has also been described for rhinovirus 2 when the cellular medium was acidified (36, 37). This manipulation allowed the authors to investigate the impact of membrane potential on virus uncoating independently of pH gradient (36).

Little is known about the diversity of signaling pathways and compartments involved in virion internalization and trafficking in enteroviruses, although a few examples have been characterized (38–44). CRISPR-Cas9 screens for enterovirus host-dependence factors routinely uncover classes of proteins involved in membrane trafficking, such as Rab GTPases, which may be related to entry. However, the specific proteins required are rarely reproducible between cell lines, suggesting that virion trafficking depends on a distinct set of proteins in each cell type (45–48). All in all, attachment, endocytosis, and viral trafficking are still active areas of enterovirus research, and general conclusions cannot be drawn about these processes.

## UNCOATING CUES AND A-PARTICLE CONVERSION

The mature virion undergoes a transition to the A-particle state in response to uncoating cues. Human enteroviruses are remarkably diverse in their use of uncoating cues. Most enteroviruses—including all enteroviruses transmitted by the fecal-oral route characterized thus far—require a cellular protein, the uncoating receptor, to catalyze the transition from native to A-particle states (49). Many enteroviruses also use low pH as an uncoating cue, either alone or in conjunction with an uncoating receptor (26, 50–53). There may be additional, undescribed cues or chemical gradients encountered during infection which are important for uncoating in a cellular setting (54). The balance of uncoating cues can be altered through single amino acid substitutions during laboratory passage experiments and may vary between isolates of a given type (33, 55–60).

For those enteroviruses dependent on uncoating receptors, evidence obtained through monovalent stimulation of the virion with membrane-bound receptors suggests that A-particle conversion occurs locally at first as individual receptor-binding sites are stimulated (61). The local release of pocket factor, the N-terminus of VP1 (hereafter, VP1_N_), and VP4 causes global redistribution of internal VP4 and possibly restructuring of the viral genome (61). Cryo-EM studies of A-particles suggest a heterogeneous composition with partial VP4 occupancy and VP1_N_ possibly assuming multiple conformational states (62, 63). VP4, together with VP1_N_, are the membrane-active components of enterovirus virions. The A-particle is expanded radially by ~4% relative to the mature virion (30, 61). The loss of pocket factor primes the particle for loss of VP4 and externalization of VP1_N_, which makes the particle more hydrophobic, another distinguishing characteristic of the A-particle (64). Poliovirus A-particles are still infectious (about 10^3^- to 10^5^-fold less infectious than mature virions), despite lacking poliovirus receptor-binding activity and having partially lost VP4 (65, 66). After release of the genome, the A-particle becomes an empty capsid (Fig. 1F). The transition from mature virions to empty capsids can also be facilitated by heating mature virions (30, 67).

## MODELS FOR GENOME RELEASE

The final step of enterovirus entry, release of the genome from the A-particle and into the cytosol, has not been directly observed. Likewise, metastable intermediates between A-particle formation and complete genome release have not been identified. Triggering genome release may also require interactions with a cellular endomembrane and/or other cellular factors, preventing direct observation of genome release in simple *in vitro* systems (68). For these reasons, our understanding of the genome release process lags behind other areas of the biology of enterovirus entry.

**Fig 2:**
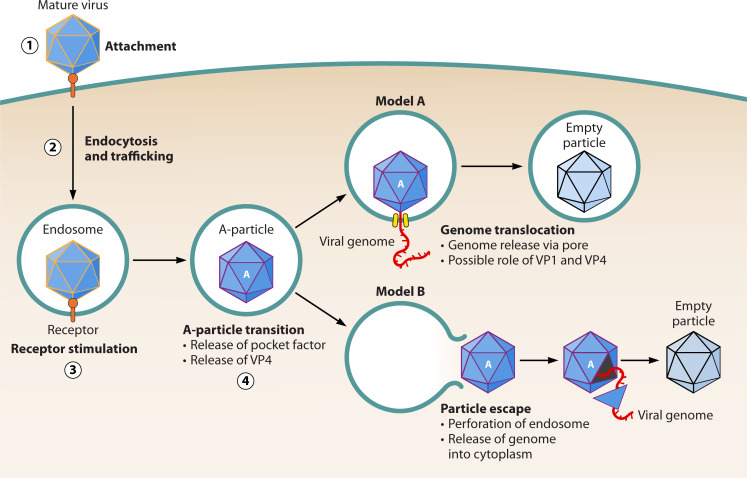
Summary of the *Enterovirus* entry process. (1) Attachment, (2) endocytosis and trafficking, (3) receptor stimulation, and (4) A-particle transition. Two models for genome release are depicted: model A is the “genome translocation” mode; model B is the “particle escape” model.

Here, we formalize two competing models for genome release (Fig. 2). The most widely accepted model is the “genome translocation model.” Under this model, a combination of viral proteins form a structure through which the genome passes from the particle and into the cytosol; endosome penetration and genome release are coupled. This structure is thought to be composed of some combination of VP4 and VP1_N_. In poliovirus, the genome appears to be released from the particle close to the quasi-threefold axis (69, 70). The particle remains anchored to the pore or to the endosomal membrane in the vicinity of the pore, shielding the viral RNA from degradation by RNases (71). The alternative model, which we call the “particle escape model,” posits that the virion or A-particle crosses the endosomal membrane and releases its genome in the cytosol. This process would be analogous to Reovirus or Adenovirus endosome escape, with endosome penetration and particle uncoating separated into two steps (72, 73). Both processes have been demonstrated in cell-free *in vitro* systems, but neither has been directly shown to initiate infections in cells.

## SUPPORT FOR THE GENOME TRANSLOCATION MODEL

The genome translocation model makes several predictions:

1. The viral structural proteins form size-selective pores in membranes that facilitate passage of the genome across the membrane.
2. The genome is not exposed to the endosomal lumen during translocation.
3. The genome exits from the particle in an ordered manner that involves breaking of RNA secondary structures.
4. The particle remains associated with the endosome after delivery of the genome to the cytosol.

Prediction 1 is partially supported by the available evidence; predictions 2–4 have strong support.

One biochemical characteristic of the 135S particle is increased hydrophobicity. This results from the increased externalization of the hydrophobic internal sequences VP4 and VP1_N_. These regions are targets for antibodies despite being internal to the mature virion (21, 22, 64, 74). The N-terminus of VP1 contains a ~20 amino acid amphipathic helix in all enteroviruses and is responsible for poliovirus liposome binding *in vitro* (64). VP4 and, to a lesser extent, VP1_N_ can also permeabilize liposomes *in vitro* (74–77). The entry process also induces endosome permeabilization in cells, although detailed mutational studies have not been performed to identify which protein domains are responsible (78, 79).

Small deletions in the VP1_N_ peptide (Δ1–4 and Δ8–9) cause defects in poliovirus entry (64, 80). Similarly, mutations in VP4 at the conserved threonine 28 abolish or delay uncoating (81, 82). These mutations also altered the ion channel-forming properties of VP4. The membrane-disrupting property of VP4 maps to the N-terminal 2/3 of the protein, which is consistent with increased evolutionary constraint in this region (83). Supporting the hypothesis that VP4 forms a multimeric pore during uncoating, VP4 oligomerized *in vitro* in the presence and absence of lipids (76). However, there remains no direct evidence that VP4 can translocate RNA across a membrane. VP4 is disordered in solution and possibly in the context of membranes as well, which has hampered our understanding of this peptide (84).

The RNA translocation process appears to shield the RNA from the endosomal lumen. Exogenous RNase added at high concentrations was unable to cleave RNA from any internalized poliovirus particles, whether or not they underwent uncoating, and did not have an effect on their infectivity (71). This was the case even when RNase was directly chemically conjugated to the viral particle. This study definitively ruled out models in which RNA is released in the endosome, although the particle escape model is not ruled out by this evidence.

Genome release is directional, beginning with the 3′ end of the genome (68, 85). In studies of rhinovirus 2, about 700 nucleotides at the 3′ end of the genome were released after pH-mediated A-particle conversion, but this process did not advance any further even after overnight incubation. However, in cells, or *in vitro,* when a membranous cellular fraction from HeLa cells was added, the genome was released within minutes. In microscopy studies, protein was degraded after incubation, but the RNA signal persisted, suggesting that capsid protein is transported to lysosomes and degraded after release of the encapsidated genome (86). Moreover, genome release appears to break RNA secondary structures because RNA-intercalating dyes and neutral red dissociate from the genome as it is released (35).

## SUPPORT FOR THE PARTICLE ESCAPE MODEL

The particle escape model makes relatively fewer predictions than the genome translocation model. This model requires (i) that the endosome be disrupted sufficiently for the diffusion of the viral particle across the endosomal membrane and (ii) that the particle be able to release its genome without the presence of a cellular endomembrane. There is evidence to support both predictions. However, the particle escape model fails to explain long-established aspects of enterovirus biology, such as the role of VP1_N_ and VP4, which this model bypasses entirely.

## WHAT HAPPENS TO THE ENDOSOME?

One element that partially differentiates the two models of uncoating is the fate of the endosomal membrane. The release of the genome from a sealed endosome precludes the particle escape model but is not necessarily required for the genome translocation model. The experimental record is mixed on the presence and extent of endosome disruption. *In vitro* studies using rhinovirus type 2 showed that virion-loaded endosomes isolated from infected cells do not release cointernalized biotin-dextran (78). Further studies using a reverse transcriptase-encapsulating liposome model found that these liposomes remain sealed at lower virion concentrations but can release their contents at higher concentrations (87).

The phospholipase PLA2G16 was found to play a critical role in enterovirus uncoating across multiple cell types and virus types (rhinoviruses were not tested) (45, 55). PLA2G16 is recruited to endosomes by non-microbial membrane damage as well as by virus infection. Cells defective in galectin-mediated membrane damage sensing or autophagy no longer required PLA2G16 to become infected, indicating that cellular membrane damage-sensing processes impose a requirement for a short window between membrane damage and genome release (88). Importantly, the enzymatic activity of PLA2G16 was required.

## MEASURING ENTRY KINETICS IN CELLS AND *IN VITRO*

The most common method used to measure entry time in enteroviruses uses light-sensitive virus grown in the presence of neutral red (89, 90). Genomes that have been released from such virus particles are no longer light sensitive. At various time points after initiating an infection, infected cells are irradiated with white light. The amount of infectivity remaining after irradiating at different time points can be used as a readout of entry kinetics. This assay has been used to determine a *t*_1/2_ value for poliovirus uncoating (30, 35), and to characterize defects in entry caused by mutations in poliovirus (80, 81).

Other methods to measure entry kinetics are based on dual labeling of the encapsidated viral RNA and viral capsid protein (35). In this case, total internal reflectance fluorescence (TIRF) microscopy was used to extract kinetic parameters from individual poliovirus entry events in live cells. Poliovirus uncoats near the cell surface rapidly after endocytosis and therefore remains within the evanescent field observable by TIRF; this assay may not be directly portable to other enteroviruses, depending on where in the cell they undergo uncoating. Entry time measurements by this method were in agreement with those obtained by neutral red inactivation.

More recently, a novel TIRF-based assay was developed to monitor genome translocation *in vitro* by individual viral particles (91). Here, receptor- and biotin-decorated liposomes were attached to a streptavidin-coated glass slide. These liposomes contain dyes which increase in fluorescence when they bind to RNA, allowing observation of fluorescence traces for individual poliovirus virions in the process of translocating their genomes into liposomes. In this system, genome translocation was inferred to take an average of 3.7 minutes, which is similar to previous estimates of 2 minutes for rhinovirus A2 in cells (68).

## STRUCTURAL BIOLOGY APPROACHES

Our view of enterovirus uncoating has developed through structural biology studies beginning with the crystal structures of rhinovirus 14 and poliovirus 1 (92, 93). Cryo-electron microscopy now enables the routine determination of structures of enterovirus mature virions, A-particles, receptor-bound intermediates, and empty capsids; a number of such structures have been solved over the past 10 years across the genus (11, 14, 30, 94–105). Recent studies have also begun to use single-particle analysis to resolve interactions between the viral RNA and the capsid in the mature virion, an underexplored area of research (106–108).

Since uncoating appears to involve interactions with a cellular endomembrane, structural biologists studying enteroviruses have also incorporated membranes in *in vitro* systems to study uncoating (61, 109–112). Strauss et al. discovered the presence of a density connecting the viral particle to liposome membranes using a receptor-decorated liposome model (110). Concurrently, similar studies using rhinovirus 2 found a particle in close apposition with a membrane, binding over a twofold axis of symmetry (111). Subsequent studies used nanodiscs to present the receptor to the virus in a more native-like context, again finding a direct, though undefined, interaction between the virus and the membrane, independent of the receptor (61, 112).

Recently, cryo-electron tomography was used to observe genome translocation in enteroviruses (113). In this study, particles were not associated with membranes, let alone actively translocating RNA, in any of the four enteroviruses tested. Given that uncoating is estimated from *in vitro* experiments to take place on the order of minutes (91), this poses a challenge to the genome translocation model of uncoating. Particularly challenging was the assertion that a natural cellular process of endosome damage was responsible for the rupture, rather than an activity intrinsic to the virus. Under this model, disassembly of the viral particle and release of the genome (114, 115) could enable virions that escape endosomes to uncoat in the cytosol. This disassembly was observed in single-particle cryo-EM analysis of echovirus 18 and studied through molecular dynamics simulations. The tomography study used a high concentration of particles and was not able to assess which of the observed particles could initiate productive infection. This study highlights several challenges inherent in studying the uncoating process in a native setting, including the potential for unproductive infection events, the possible influence of MOI, and the transient nature of the genome release event.

## KEY EXPERIMENTS TO SUPPORT EACH MODEL

Under the particle escape model, genome release is not coupled to endosome perforation. Therefore, the particle escape model implies that endosome perforation can be supplied in *trans* to facilitate infection, with VP4 and VP1_N_ membrane activity not strictly required to initiate an infection. This has not been tested, but such experiments would be straightforward. For example, sterile membrane damage can be induced using hypotonic shock or agents such as polyethyleneimine, and membrane damage can also be provided in *trans* using other non-enveloped viruses such as reovirus. If mutants attenuated in membrane activity, such as VP4 T28G, can productively uncoat with the aid of non-enteroviral membrane damage, it would suggest that membrane damage alone is sufficient for genome release, rather than the formation of pores by the virus *per se*. Importantly, this would mean that translocation of the genome through a pore formed by viral structural proteins is not required to explain productive genome release.

Further supporting the genome translocation model requires a careful dissection of the roles of the viral structural proteins in cells. Much of the evidence used to support this model comes from *in vitro* studies on the biochemical functions of the viral structural proteins. While individual mutants within VP4 and VP1_N_ have been isolated, the roles of these proteins have not been systematically functionally dissected. For example, do VP1_N_ mutants diminish A-particle binding to liposomes, delivery of VP4 to the liposome membrane, time to initiate genome translocation, or total genome translocation time? Such studies are now possible, thanks to tools developed over the last decade, but an effort to link *in vitro* activity to virus behavior in cells is needed. A combination of cellular assays, including neutral red and endosome damage-based biosensors, and *in vitro* assays such as liposome binding, liposome permeabilization, and TIRF-based *in vitro* uncoating should be used to analyze mutants in the viral structural proteins and dissect their roles in uncoating. Advocates of the genome translocation model frequently claim that pores composed of VP4 can translocate RNA, but insufficient evidence supports this claim. Definitively establishing the function of VP4 or the composition of the RNA-translocation complex would cement the genome translocation model, satisfying the only unsatisfied prediction made by this model.

## ARE BOTH MODELS “CORRECT”?

It is possible that multiple pathways to productive uncoating exist. The virion must be able to initiate infection under a number of conditions: different methods of transmission within- and between-hosts might establish different MOI regimes for infection. In a high MOI regime, it is more likely that a particle is in the vicinity of uninduced endosomal ruptures and can diffuse from the endosome before the rupture is repaired. Different MOIs may also deliver varying quantities of VP4 to the endosomal membrane, which may alter its ability to translocate virions or RNA. Since the particle:pfu ratio for enteroviruses tends to be on the order of hundreds to thousands (30), the infectious unit may involve more than one mature virion and more VP4 than that found within a single virion. VP4 was shown to form fibrillar aggregates at high concentrations and discrete pores under lower concentrations in detergent micelles (76). Exogenously added peptides derived from VP4 also increased the extent of genome translocation *in vitro* (91). Depending on the rate of genome (or virion) translocation in each respective pathway, uncoating by endosome disruption and particle escape may be more or less productive in different environments. It is also possible that a single universal uncoating pathway does not exist and that genome translocation and particle escape are preferentially utilized by different enterovirus types. Studies on rhinovirus 14 and rhinovirus 2 suggested that rhinovirus 14 entry involves endosome disruption, but rhinovirus 2 does not (79, 116). As an orthogonal method to assess endosome disruption, it would be interesting to test whether or not rhinovirus 2 entry results in galectin recognition of damaged endosomes or requires PLA2G16 to uncoat efficiently.

## NOVEL STRUCTURAL BIOLOGY APPROACHES

Recent advances in nanodisc technology have made it possible to produce stable nanodiscs of a range of sizes (117). It should be possible to readily test a variety of enterovirus-receptor pairs, buffer conditions, and exogenous cofactors, hopefully identifying a model system for single-particle analysis of genome release intermediates. HIV-1 virions pseudotyped with viral receptors might be another useful system to study virus-membrane interactions; this approach was recently used to identify SARS-CoV2 fusion intermediates (118).

Observing a virion in the act of translocating its RNA across a membrane in cells would provide definitive support for the genome translocation model, but as this event is transient and occurs in a very tiny fraction of an infected cell, this is a difficult task. Cryogenic correlative light and electron microscopy coupled with focused ion beam (FIB) milling may solve this problem. Fluorescent fusions of the carbohydrate recognition domain of galectin 8 (which detects endosome damage) could be used to locate endosome damage events at lower-MOI regimes, followed by FIB milling to access the site of uncoating in electron micrographs. A similar approach employing fluorescent HIV-1 cores was used to observe the process of capsid nuclear import in permeabilized T cells (119, 120). The isolation of variants with specific defects in RNA translocation may also aid in observing this event.

Other structural approaches more closely approximating the situation in cells may also be used. Endosomes have been efficiently isolated for proteomic studies using immunoprecipitation (121). A similar approach could be used to study enterovirus uncoating. Fluorescently-labeled viruses could be used to sort virus-loaded endosomes from empty endosomes for *in vitro* uncoating studies using native cellular material, or for cryo-electron tomography studies.

## OUTLOOK

Despite decades of active research, enterovirus uncoating remains an unsolved problem. Nevertheless, we believe the experimental tools are in place to connect findings made across the genus in support of one or more models of genome release. We emphasize the variety of experimental systems available to answer questions in enterovirus uncoating. The genus *Enterovirus* is highly diverse in terms of requirements for pH, cellular compartments for uncoating, uncoating receptors, and temperature. Enterovirus researchers have likewise developed a great variety of biochemical, cell-biological, biophysical, genetic and structural biology approaches during the long history of research on enterovirus uncoating. All that remains is to coordinate across this territory to solve the mystery of enterovirus genome release.
